# RETRACTION: Impact of Ileostomy on Postoperative Wound Complications in Patients after Laparoscopic Rectal Cancer Surgery: A Meta‐Analysis

**DOI:** 10.1111/iwj.70394

**Published:** 2025-03-21

## Abstract

**RETRACTION:** YangS. , LinY. , ZhongW. , XuW. , HuangZ. , CaiS. , ChenW. , and ZhangB. , “Impact of Ileostomy on Postoperative Wound Complications in Patients after Laparoscopic Rectal Cancer Surgery: A Meta‐Analysis,” International Wound Journal 21, no. 3 (2024): e14493, 10.1111/iwj.14493.37989718 PMC10898402

The above article, published online on 21 November 2023, in Wiley Online Library (http://onlinelibrary.wiley.com/), has been retracted by agreement between the journal Editor in Chief, Professor Keith Harding; and John Wiley & Sons Ltd. Following an investigation by the publisher, all parties have concluded that this article was accepted solely on the basis of a compromised peer review process. The editors have therefore decided to retract the article. The authors did not respond to our notice regarding the retraction.

**RETRACTION:** S. Yang , Y. Lin , W. Zhong , W. Xu , Z. Huang , S. Cai , W. Chen , and B. Zhang , “Impact of Ileostomy on Postoperative Wound Complications in Patients after Laparoscopic Rectal Cancer Surgery: A Meta‐Analysis,” International Wound Journal 21, no. 3 (2024): e14493, 10.1111/iwj.14493.37989718 PMC10898402

The above article, published online on 21 November 2023, in Wiley Online Library (http://onlinelibrary.wiley.com/), has been retracted by agreement between the journal Editor in Chief, Professor Keith Harding; and John Wiley & Sons Ltd. Following an investigation by the publisher, all parties have concluded that this article was accepted solely on the basis of a compromised peer review process. The editors have therefore decided to retract the article. The authors did not respond to our notice regarding the retraction.

